# What is a cure through gene therapy? An analysis and evaluation of the use of “cure”

**DOI:** 10.1007/s11019-024-10223-w

**Published:** 2024-08-22

**Authors:** Lieke Baas, Karina Meijer, Annelien L. Bredenoord, Rieke van der Graaf

**Affiliations:** 1 Department of Bioethics and Health Humanities, Julius Center for Health Sciences and Primary Care, University Medical Center Utrecht, Utrecht University, Utrecht, the Netherlands; 2 Department of Hematology, University Medical Center Groningen, University of Groningen, Groningen, the Netherlands; 3 Erasmus School of Philosophy, Erasmus University Rotterdam, Rotterdam, the Netherlands

**Keywords:** Health, Disease, Cure, Gene therapy, Ethics, Philosophy of medicine

## Abstract

The development of gene therapy has always come with the expectation that it will offer a cure for various disorders, of which hemophilia is a paradigm example. However, although the term is used regularly, it is unclear what exactly is meant with “cure”. Therefore, the aim of this paper is to analyse how the concept of cure is used in practice and evaluate which of the interpretations is most suitable in discussions surrounding gene therapy. We analysed how cure is used in four different medical fields where the concept raises discussion. We show that cure can be used in three different ways: cure as normalization of the body, cure as obtaining a normal life, or cure as a change in identity. We argue that since cure is a practical term, its interpretation should be context-specific and the various uses can exist simultaneously, as long as their use is suitable to the function the notion of cure plays in each of the settings. We end by highlighting three different settings in the domain of hemophilia gene therapy in which the term cure is used and explore the function(s) it serves in each setting. We conclude that in the clinical application of gene therapy, it could be better to abandon the term cure, whereas more modest and specified definitions of cure are required in the context of health resource allocation decisions and decisions on research funding.

## Introduction

The development of gene therapy has always come with the great expectation of being able to offer a cure for various disorders (Kimmelman 2010). One of the paradigm examples of this promise stems from the field of hemophilia care. Hemophilia is a congenital bleeding disorder that leads to spontaneous bleeding into muscles and joints if left untreated. Because hemophilia is a single-gene disorder with a wide therapeutic window, it has traditionally been perceived as the ideal model for validating general gene therapy principles (DiMichele et al. 2003; Gura 2001). After several decades of research, the first gene therapies for hemophilia A and B have received market authorization by the EMA and FDA. Simultaneously, further research and development of gene therapies for hemophilia is ongoing.

In contrast to many other disorders for which gene therapies are being developed, several treatment options are already available for hemophilia (Mannucci 2020; Peters and Harris 2018). As a result, the life expectancy of people living with hemophilia in resource-rich settings is approaching that of the general population (Franchini and Mannucci 2014; Hassan et al. 2021). Therefore, for people with hemophilia living in resource-rich settings, gene therapy is likely to become a personal choice in the palette of treatment options.

In the literature, several arguments in favor of the further development of gene therapies for hemophilia can be recognized. Most regularly mentioned are the expectations that it will circumvent many of the burdens of current treatment, lead to cost savings and will provide cure (Ozelo and Yamaguti-Hayakawa 2022; Pipe 2020; Spadarella et al. 2020; Valentino et al. 2023). Particularly the latter promise, the ability to provide cure, is embraced and used in headlines in both academic literature and news media discussing trial results (Gallagher 2022; van den Berg 2017).

However, it remains unclear what is meant when cure through gene therapy is promised. Several authors mention the potential of gene therapy to cure, but do not offer any definition or explanation of the concept (e.g. Nathwani et al. 2022; Perrin et al. 2019). Further, empirical research indicates that people living with hemophilia and their health care providers differ in their opinion whether a cure has been achieved after receiving gene therapy in a trial (Baas et al. 2023).

Therefore, the aim of this paper is to analyse how the concept of cure is currently used in practice and evaluate which of these uses is most suitable in discussions surrounding gene therapy for hemophilia.

## Method

The concept of cure is elaborated on rarely in the philosophical literature. However, according to its definition in the Oxford dictionary, cure is concerned with restoring health. It is therefore helpful to look at the debate on the concepts of health and disease. There is a longstanding theoretical debate on health and disease, and several competing theories have been formulated to explain their meaning and function (Boorse 1997; Nordenfelt 2007; Wakefield 1992). In more recent contributions to this debate, health and disease are no longer considered to be theoretical concepts, but practical terms, that fulfill a certain function in a given context, for instance in different academic disciplines, in different institutional settings in society or in different health practices (Haverkamp et al. 2018; Kukla 2022; Powell and Scarffe 2019; van der Linden and Schermer 2022). Because they are practical concepts, it has been argued that standard conceptual analysis is ill-suited to understand their meaning (De Vreese 2017; Kukla 2022; Powell and Scarffe 2019). It is therefore argued that instead of searching for final definitions with necessary and sufficient conditions, the debate should focus on developing multiple context-dependent definitions, that each have their own merit in a certain context or institution (Kukla 2022; Powell and Scarffe 2019; van der Linden and Schermer 2022).

Based on these insights, we will treat the notion of cure as a practical concept as well. We proceed in two steps. First, we aim to obtain insight into the differing ways in which the concept of cure is currently used in practice. We do so by examining how the concept of cure is used in various medical fields in which the concept of cure is contested and therefore regularly discussed: oncology, psychiatry, care for people with a hepatitis C virus (HCV) infection and care for d/Deaf people. These fields were chosen because of the diversity in the type of pathology they are concerned with and the variety of patient groups involved in each of the fields. We have included a variety of literature discussing cure in each of the fields, including reviews, qualitative studies, and argumentative papers.

Second, we turn to the debate on gene therapy for hemophilia. After, we apply the identified uses of cure to the debate on gene therapy for hemophilia. We then continue by distinguishing three different settings in which the notion of cure is used in relation to gene therapy and evaluate anddiscuss factors that are important to take into consideration when using the concept of cure in a certain setting. Accordingly, we evaluate which of the interpretations of cure is most suitable.

## Use of the concept cure

The term “cure” is used regularly, although it is often left undefined. However, based on a review of uses of cure in the literature on the abovementioned fields and discussion within the research team, we identified three different interpretations of cure: cure as normalization of the body, cure as obtaining a normal life, cure as a change in identity. We also discuss situations in which the use of cure is deliberately avoided.

### Cure as normalization of the body

When an explicit definition of cure is provided in the medical literature, it often refers to cure as some way of normalization of the body (e.g. Linas et al. 2014; Mason and Dunnill 2008; Tralongo et al. 2019). An example of this is the promise of cure through regenerative medicine, as regenerative medicine promises to “replac[e] or regenerat[e] human cells, tissues or organs, to restore or establish normal function” (Mason and Dunnill 2008, p. 4).

Many of these definitions have an instrumental purpose, as they are developed to be able to compare the outcomes of different treatment options. Therefore, they incorporate an element of temporality, whereby cure is defined and assessed over a certain period of time after treatment. For instance, in oncology, cure can be defined as no return of cancer for at least five years after treatment (Tralongo et al. 2019). Similarly, in the treatment of HCV infection, cure is often defined as sustained virological response (SVR) at 24 weeks after treatment (Linas et al. 2014; Pearlman and Traub 2011).

The definitions of cure mentioned above are applicable to the level of the individual patient. In contrast, cure can also be defined on a population level. This use is also common in oncology, where a “statistical cure” for cancer “implies that the mortality rate of people diagnosed with a certain neoplasm returns to the level expected in the general population of the same sex and age, or, equivalently, when the excess mortality rate approaches zero” (Tralongo et al. 2019, p. 2).

The idea of normalization of the body has received criticism from the field of disability studies, where it is argued that the focus on normalizing bodies does not value the lives and bodies of disabled people (Beauchamp-Pryor 2011).

### Cure as obtaining a normal life

Cure is also used to describe a normalization of various aspects of daily life. This idea recurs often in literature on patient experiences and wishes of treatment for HCV infection, in particular HCV obtained through injective drug use (Harris 2017; Kagan et al. 2023; Madden et al. 2018). It is argued that in general, cure is understood to mean an end to illness and its effects, as well as restoration of a prior state of health and well-being, as a result of which cure is seen as an endpoint (Kagan et al. 2023). Many people with HCV express the hope that a cure will allow for a return to normality (Harris 2017; Madden et al. 2018). Because globally, people who inject drugs and people in prison are the two main groups affected by HCV, overcoming social stigma is an important part of cure for these patients (Harris 2017; Oru et al. 2021). However, this understanding of cure is criticized, because the focus of cure as end-point overlooks the ongoing experiences, needs and suffering of people after the completion of treatment (Kagan et al. 2023).

In oncology literature there is also reference to “our shared belief about what a cure really is”, namely “the idea that at some point after treatment, one’s life is no different than someone who had not experienced that disease” (Prasad 2014, p. 478). Further, in the fields of both oncology and HCV treatment, it is also recognized that the ability to obtain a normal life also relates to laws, policies and social structures, such as access to insurance or governmental benefits, and cannot be solely achieved by biomedical cure (Dumas et al. 2016; Kagan et al. 2023; Miller et al. 2013; Seear et al. 2023).

### Cure as a change in identity

Finally, cure is conceived as a change in identity focusing on how both the illness experience and the transition to a state of health can become integrated into a person’s narrative identity. This interpretation can be recognized in qualitative research on experiences of people with an HCV infection. This research indicates that many people, especially people who contracted the infection through injecting drugs, value a cure for several outcomes other than “sustained virological response”, such as its impacts on identity and social connections (Madden et al. 2018). As the authors of the study define cure as sustained virological response, they describe the other outcomes as “beyond cure” (Madden et al. 2018). This group of patients in particular hopes that clearing the HCV infection would lead to a positive change in identity, which would also help them to leave the label and stigma of “drug user” behind. Therefore, for several people living with HCV infection a cure would not mean a “return to normality”, but a “reawakening” or “rebirth” (Harris 2017). As a result of these high expectations, some people were disappointed by the effects of a cure for HCV infection, as they thought it did not live up to the promises and hype (Madden et al. 2018).

Similarly, it has been argued that the distinction between between technology that is assistive and curative does not arise from the form or function of the technology, but from the role the technology plays in establishing the user’s relational narrative identity as either belonging to the social group of people with a disability or disease or the social group of people without a disability or disease (Stramondo 2019, p. 1128).

The discussion surrounding a change in identity also recurs among people with various disabilities. For instance, for some d/Deaf people, deafness is seen as part of who they are and the social group to which they belong (McIlroy and Storbeck 2011). Therefore, some consider the idea of cure for deafness undesirable (Stramondo 2019).

### Alternatives to cure

In both the fields of psychiatry and oncology, some authors argue that the notion of cure should be abandoned, as it is thought that a cure is unreachable and that the focus on cure is not beneficial for the quality of care. For instance, in psychiatry, there is a growing movement arguing that mental illnesses are seldomly cured, and should be considered “vulnerabilities” rather than “diseases” (van Os et al. 2019). Instead of focusing on cure, it is argued that psychiatric care should focus on “recovery” or “healing” (Barber 2012; Kirmayer 2004; Slade et al. 2014; van Os et al. 2019). These concepts are not focused on “getting better” or “becoming normal”, but mainly focus on well-being, resilience, and the ability to adjust to and manage challenges, which can be achieved by having attention for the relational, narrative, ritual and metaphorical aspects of care (Egnew 2005; Hinton and Kirmayer 2017; Kirmayer 2004; Slade et al. 2014; van Os et al. 2019; Wampold 2015).

Similarly, in oncology, although the term cure is used abundantly in the literature (Prasad 2014), in practice, many oncologists are hesitant to tell a patient they are cured, because of a lack of confidence about the risk of relapse (Corn et al. 2020; Miller et al. 2013; Sharpless 2018). Instead, terms like “in remission” or “no evidence of disease” are used in cancer care (Miller et al. 2013). Furthermore, without the possibility of being able to speak of “cure”, people living with a history of cancer often choose to speak of “cancer survivorship”, as they prefer not to be called patients (Astrow 2012). The term cancer survivorship is used from the time of diagnosis throughout the rest of life. The concept draws attention to the phases of care required after active cancer treatment and also includes the health effects and emotional toll of a cancer diagnosis on family members, friends and caregivers (Shapiro 2018).

## Cure for hemophilia

Although the concept of cure is often used without being defined in care for hemophilia, each of the abovementioned uses of cure can be recognized in the field. To start with the most stringent definition of cure: “complete correction of previous bleeding tendency with normalized clotting factor levels 5 years after curative treatment, requiring no further treatment (with coagulation factor or other treatments), not even for surgery or bleeding. Cure is phenotypically intended and does not include: eliminating transmission of hemophilia to children or fully reverting established damage” (van Balen et al. 2021). This definition is focused on normalization of the body, and, as the definition was developed to be able to compare outcomes of different treatment options, also incorporates an element of time (van Balen et al. 2021). Similar definitions of normalization of the body through the criteria of elimination of bleeding and not requiring prophylactic treatment can also be recognized in other uses of the term cure (van den Berg 2017).

Simultaneously, cure is also used to refer to obtaining a normal life. For instance, Skinner et al. argue that there are several “interrelated cure issues”, that also entail lost education and career opportunities and financial consequences (2004, p.115). Similarly, the concept of a “hemophilia-free mind”, defined as the absence of psychological burden and permanent thoughts about hemophilia and its complications, has been suggested as a goal for further development of hemophilia treatment (Hermans and Pierce 2023; Krumb and Hermans 2021). It has been argued that the concept of a hemophilia-free mind should also be applied to family members taking care of or living with people with hemophilia (Hermans et al. 2024). Although the authors do not relate a “hemophilia-free mind” to the concept of cure, the idea of not having to think about a disorder is part of having a life unaffected by the disorder, comparable to how a cure as obtaining a normal life is understood.

Finally, the idea of cure as establishing a change in identity can also be recognized. In the literature on hemophilia gene therapy, cure as establishing a change in identity is discussed in accounts of people living with hemophilia who do not desire to receive gene therapy because they do not want to lose the identity of being a person with hemophilia (Fletcher et al. 2021; Zhang 2018). For these individuals, the notion of cure as corresponding to a change in identity is undesirable.

In the discussion surrounding hemophilia, we did not encounter any arguments in favor of abandoning the term cure or proposing alternatives to use in its place.

## Gene therapy and the role of cure

In this section we highlight three different settings in which the term cure is used in relation to gene therapies, particularly gene therapy for hemophilia, and look at the role the concept can play in each of these settings. We do not claim to provide an exhaustive overview of the different settings and practices in which the concept is used, but provide a starting point for further thinking about the role and function of the concept of cure in relation to gene therapies.

### Cure in the clinical application of gene therapy

One evident context in which the term cure is used is in a clinical setting, where patients may receive gene therapy as part of research or care. “Cure” can here be used as a goal for or outcome of treatment. In a setting in which people decide whether to opt for gene therapy, it is important that they have the right information to make an informed choice. Therefore, use of the word cure, or potential cure, as gene therapy is often described, may create wrong expectations if it is not clear what is meant with the term, or if patients attach a different meaning to the word than their physicians do.

In both clinical care and research, specific endpoints are defined in order to be able to measure effectiveness. Often, such endpoints do not include a more abstract notion such as “cure”. Nonetheless, the definition of cure by Van Balen and colleagues, mentioned in Sect. 4, is aimed to be used as a standard in outcome. However, as the definition explicitly excludes eliminating transmission to offspring or reverting established damage, it might not live up to the expectations of people living with hemophilia who understand cure to mean a normalization of their life or a change in identity.

This indicates that cure may have a different meaning for various people with hemophilia. This is also supported by qualitative research we have conducted. Our interview study that clinicians and people with hemophilia sometimes differ in their opinion on whether people are cured after gene therapy, and that some people with hemophilia have expectations that are unrealistically high according to their physician (Baas et al. 2023). The term is thus used ambiguously in the field, which could lead to unclarity between stakeholders or in conversations between people with hemophilia and their health care providers.

Furthermore, after having received gene therapy and potentially having obtained normalized coagulation levels and bleeding symptoms, which could be considered a form of cure as “normalization of the body”, many people with hemophilia will still experience complications from the disorder. People living with hemophilia often have joint damage resulting from earlier bleeding, which will progress and continue to impact quality of life (van Vulpen et al. 2018). Similarly, because hemophilia is a congenital disorder, it can still be passed on to future generations, people living with hemophilia have only ever known life with hemophilia, and usually they have several stories of living with hemophilia among their relatives. Therefore, no longer having hemophilia will never be a “restoration” of a previous state of health, but will likely resemble a state of being in which people do not have to worry about bleeding, but still live with the consequences and previous experiences of living with hemophilia will remain.

Therefore, in interactions between people with hemophilia and health care providers, it might be desirable to refrain from using the term cure, and instead opt for merely using specific endpoints and effects on quality of life when discussing potential benefits of a treatment or trial. Furthermore, because people may still need some form of medical care after gene therapy and it is uncertain whether gene therapy has a long-lasting effect, a term that describes life after treatment for disease, similar to the role the term “cancer survivorship” plays, could be most suitable to describe the period after having received gene therapy. This could prevent confusion and thereby improve informed decision making, as well as be a more accurate description of the life and care required after gene therapy. Therefore, in the patient-physician relationship, the physician should become aware of the patient’s expectations of what a cure entails and what gene therapy can achieve, and critically assess to what extent this is a realistic expectation.

### Cure in resource allocation decisions on gene therapy

The concept of cure also plays a role in decisions on resource allocation with regard to gene therapy. It has been argued that having a specified concept of disease is particularly important regarding questions when medical intervention is required and justified, including to be able to distinguish between treatment and enhancement, to prevent medicalization and in questions regarding distributive justive in health care (Kukla 2022; Powell and Scarffe 2019; Schramme 2007).

Currently, the notion of cure is already used in questions regarding distributive justice. For instance, in the Netherlands, there is a policy-instrument, the Coverage Lock (CL), that is used to evaluate new, expensive medicines before they are included in basic health insurance. The EMA-approved gene therapies for hemophilia A and B are currently undergoing the CL procedure. In the CL process for another gene therapy, Libmeldy to treat metachromatic leukodystrophy, the potential to cure was considered one of several arguments that together made a higher price acceptable (Zorginstituut Nederland 2022). Similarly, in the literature it is argued that therapies with a potential to cure may have a special value that justifies a high price (Hendriks and Pearson 2021; Husereau 2015). It has been suggested that such distinctive elements of value include liberation from disease identity, liberation from stigma, and liberation from the burden of ongoing treatment (Hendriks and Pearson 2021). In our analysis, these elements are not just part of the value of a cure, but also part of the conceptualization of a cure.

In decisions on reimbursements, it is important to be explicit about what is meant with a cure and in what ways a product with a potential to cure differs from other treatment options, to assess what added value a cure may bring. If cure is defined as a change in identity, it is questionable to what extent this is valuable for people living with hemophilia, as currently, it has mainly been described as an undesirable outcome. Cure understood as normalization of the body raises several questions: should normalization also include restoring residual damage to joints? Should it include potential side effects of gene therapy? Furthermore, cure as obtaining a normal life is dependent on more factors than a treatment or medical intervention, such as persons’ opportunities in life and their mental state. This means that before any of the uses of cure is valuable in decisions on reimbursement, several questions need to be answered.

### Cure in the context of research funding decisions on gene therapy

The notion of cure is also used in trial protocols and research proposals for new gene therapies and other innovative treatments. For instance, a trial protocol mentions that gene therapy, in contrast to existing treatment options, offers the potential to cure hemophilia A (*Clinicaltrials.Gov*, 2023). Other registered hemophilia gene therapy trial protocols do not explicitly mention the promise of a cure through gene therapy, but do mention that one of the downsides of current standard treatment is lack of a cure (*ClinicalTrials.Gov*, 2019). This suggests that, although cure is not included as a study outcome of current gene therapy trials, it appears to be a goal of the program of gene therapy. As trials employ specific endpoints in order to measure effectiveness, if cure would be included as an outcome measure for a trial, it would have to be defined specifically and with a component of time (e.g. five years) in order to be able to serve as an endpoint to evaluate the effectiveness of an intervention. This suggests that cure as form of normalization of the body might be most suitable. Further, cure as obtaining a normal life or a change in identity relates more to quality of life, which is often used as a secondary endpoint in trials. If cure is considered to be one of the primary goals of gene therapy, it raises the question whether quality of life should also become a primary endpoint of trials rather than a secondary endpoint.

In contrast, in the development of research proposals, the notion of cure is often used as a more abstract promise of what the study will achieve. While working on research proposals, researchers regularly have to boost expectations in order to attract funding (Oerlemans et al. 2014). As a result, the term cure can contribute to hype and high expectations surrounding gene therapy, which may increase further when cure is ill-defined or interpreted differently by various stakeholders. Therefore, although use of the term can be valuable in this setting, it is important to be hesitant when translating these promises to other settings.

## Conclusion and future directions

The promise of cure appears to be closely tied to expectations of gene therapy. Simultaneously, the term often remains undefined or is used to refer to varying outcomes. Therefore, we analysed how the term is used in several medical fields. We have shown that cure can be used to refer to at least three different outcomes of treatment: normalization of the body, obtaining a normal life, and a change in identity. We have argued that since cure is a practical term, its interpretation should be context-specific and the various uses can exist simultaneously, as long as their use is suitable to the function the notion of cure plays in each of the settings. Without aiming to be exhaustive, we then highlighted three contexts in which the term cure is used. We argue that in the clinical application of gene therapy, it could be better to abandon the term cure, whereas a more modest and specified definition of cure is required in the context of health resource allocation decisions and decisions on research funding.

These considerations on the use of cure can provide more clarity and stimulate reflection in discussions surrounding gene therapies or other innovative treatments. Moreover, awareness of the various potential interpretations of cure should urge for carefulness when speaking of “cure”, especially when the contexts in which the term is used meet or when stakeholders transcend the context in which they usually operate, as incongruent use of cure may cause misunderstanding and potentially unrealistic expectations.

This paper has provided insight into various ways in which the term cure can be understood and has argued that depending on the context, different interpretations might be more or less valuable. Thereby, the paper invites further deliberation on the contexts in which the term cure is used and provides input for the discussion surrounding the benefits of gene therapy.
